# Characterization of the chloroplast genome sequence of *Bonia amplexicaulis* (L.C.Chia, H.L.Fung & Y.L.Yang) N.H.Xia (Poaceae)

**DOI:** 10.1080/23802359.2021.1972871

**Published:** 2021-08-31

**Authors:** Li-Ying Feng, Li-Zhi Gao

**Affiliations:** Institution of Genomics and Bioinformatics, South China Agricultural University, Guangzhou, China

**Keywords:** *Bonia amplexicaulis*, chloroplast genome, Poaceae, phylogeny

## Abstract

*Bonia amplexicaulis* (L.C.Chia, H.L.Fung & Y.L.Yang) N.H.Xia is a member of the Bambusoideae subfamily in Poaceae. In this study, we sequenced, assembled and characterized the complete chloroplast genome of *B. amplexicaulis.* The complete chloroplast genome was 139,935 bp in size, including a large single copy region of 83,453 bp, a small single-copy region of 12,860 bp and a pair of reverse repeats of 21,811 bp in size. The annotation of the *B. amplexicaulis* chloroplast genome indicates that it contained 83 protein-coding genes, 36 tRNA genes and 8 rRNA genes. Our phylogenetic analysis of all protein-coding genes from the 36 complete chroloplast grass genomes using *Cyperus rotundus* as outgroup showed that *B. amplexicaulis* is closely related to *Otatea glauca* and *Pariana campestris* to form the Bambusoideae lineage of the grass family.

*Bonia amplexicaulis* Chia belongs to the Bambusoideae subfamily of Poaceae. It is a rare and important cash crop with good growth in limestone mountain areas, widely distributed in the rocky desertification areas of Guangxi, Guangdong, Guizhou, Yunnan and other provinces of China (Xia 1996). Its root system is well developed and adheres to the soil of rock mountain, which is of great significance to the ecological protection and management in the Shidesert area (Xia 1996). Poaceae is the fifth largest flowering plant in the world and is of great economic importance (Saarela et al. 2018). Recent decades have witnessed great efforts to reconstruct the phylogeny of Poaceae. However, the phylogenetic relationships within several clades have not been fully resolved (Clark et al. 1995). Compared with the nuclear genome, the chloroplast genome is inherited matriarchally, the sequence is relatively conservative and the genome is small. Recent decades have witnessed rapid progress in comparative chloroplast genomics that has been widely applied to studying plant phylogenetics and evolution (Huang et al. 2014; Gao et al. 2019). Thus, it is needed to further generate a large number of grass complete chloroplast genomes toward a well-resolved phylogeny of the grass family.

In this study, *B. amplexicaulis* plants were collected in the suburbs of Kunming (24°26′50″N, 101°50′18″E), Yunnan Province, China. A voucher specimen was deposited at SCAU (the herbarium of the College of Agriculture, South China Agricultural University https://nxy.scau.edu.cn, Li-zhi Gao, SCAUgenomics@163.com), China, under the voucher number (SCAU 2020126). About 20 g fresh mature leaves were sampled from *B. amplexicaulis*, and cpDNAs were extracted by following a modified high salt method reported formerly (Shi et al. 2012). After the cpDNA isolation, approximately 5–10µg of DNA was sheared, followed by adapter ligation and library amplification, and then subjected to Illumina Sample Preparation Instructions. The fragmented cpDNAs were sequenced at both single-read using the Illumina Genome Analyzer IIx platform at the in-house facility at The Germplasm Bank of Wild Species in Southwestern China, Kunming, China. The obtained paired-end reads (2 × 100 bp read lengths) were assembled using SOAP *de novo* (Li et al. 2010). Regions with ambiguous alignment (conflicted reads mapped to the same genomic region) were trimmed off manually and considered as gaps. Polymerase chain reaction (PCR) amplified fragments yielded by primers derived from the terminal ends of contigs, and the fragments were then sequenced to flank the gap regions. The PCR amplification reactions were template denaturation at 80 °C for 5 min followed by 30 cycles of denaturation at 95 °C for 30 sec, primer annealing at 55 °C for 30 sec, and primer extension at 65 °C for 1 min; followed by a final extension step of 5 min at 65 °C. PCR products were separated by electrophoresis in 1.5% agarose gel and sequenced on an Applied Biosystems (ABI) 3730 automated sequencer. Subsequently, gene prediction and annotation were performed by DOGMA (Wyman et al. 2004).

The complete chloroplast genome of *B. amplexicaulis* was 139,935 bp in size, comprising two inverted repeat regions (IRs) with a total of 43,622 bp in size, which were split by a large single copy (LSC) with 83,453 bp and small single copy (SSC) with 12,860 bp in length. The chloroplast genome contained 127 functional genes, including 83 protein-coding genes, 36 tRNA genes, and 8 rRNA genes. A total of 19 genes were repeated in the IR regions, including 4 rRNA genes (*rrn16*, *rrn23*, *rrn4.5*, and *rrn5*), 7 protein-coding genes (*rps*19, *rpl2*, *rpl23*, *rps7*, *rps15*, *ycf68* and *ndh*B) and 8 tRNA genes (*trn*H*-*GUG, *trn*I*-*CAU, *trn*L*-*CAA, *trn*V*-*GAC, *trn*I*-*GAU, *trn*A*-*UGC, *trn*R*-*ACG and *trn*N*-*GUU). The overall GC content of the *B. amplexicaulis* chloroplast genome was ∼38.86% with the corresponding values of 36.96%, 33.13% and 44.19% in the LSC, SSC, and IR regions, respectively.

To determine the phylogenetic position of *B. amplexicaulis* in the grass family, 35 grass chloroplast genomes together with *Cyperus rotundus* from Cyperaceae were separately downloaded from GenBank. Phylogenomic analysis was performed by incorporating the *B. amplexicaulis* chloroplast genome obtained in this study. All sequences of protein-coding genes were aligned with MAFFT 7.409 (Katoh et al. 2002). Using *C. rotundus* as outgroup the phylogenetic tree was reconstructed by means of the maximum likelihood method implemented with RAxML (Stamatakis 2014) based on 1000 bootstrap replicates. Our results indicated that the 35 studied grass species were evidently clustered into the twelve subfamilies of Poaceae with strong bootstrap supports (Figure 1). It is apparent that *B. amplexicaulis* is closely related to *Otatea glauca* and *Pariana campestris* from Bambusoideae of the grass family with strong bootstrap supports.

**Figure 1.**
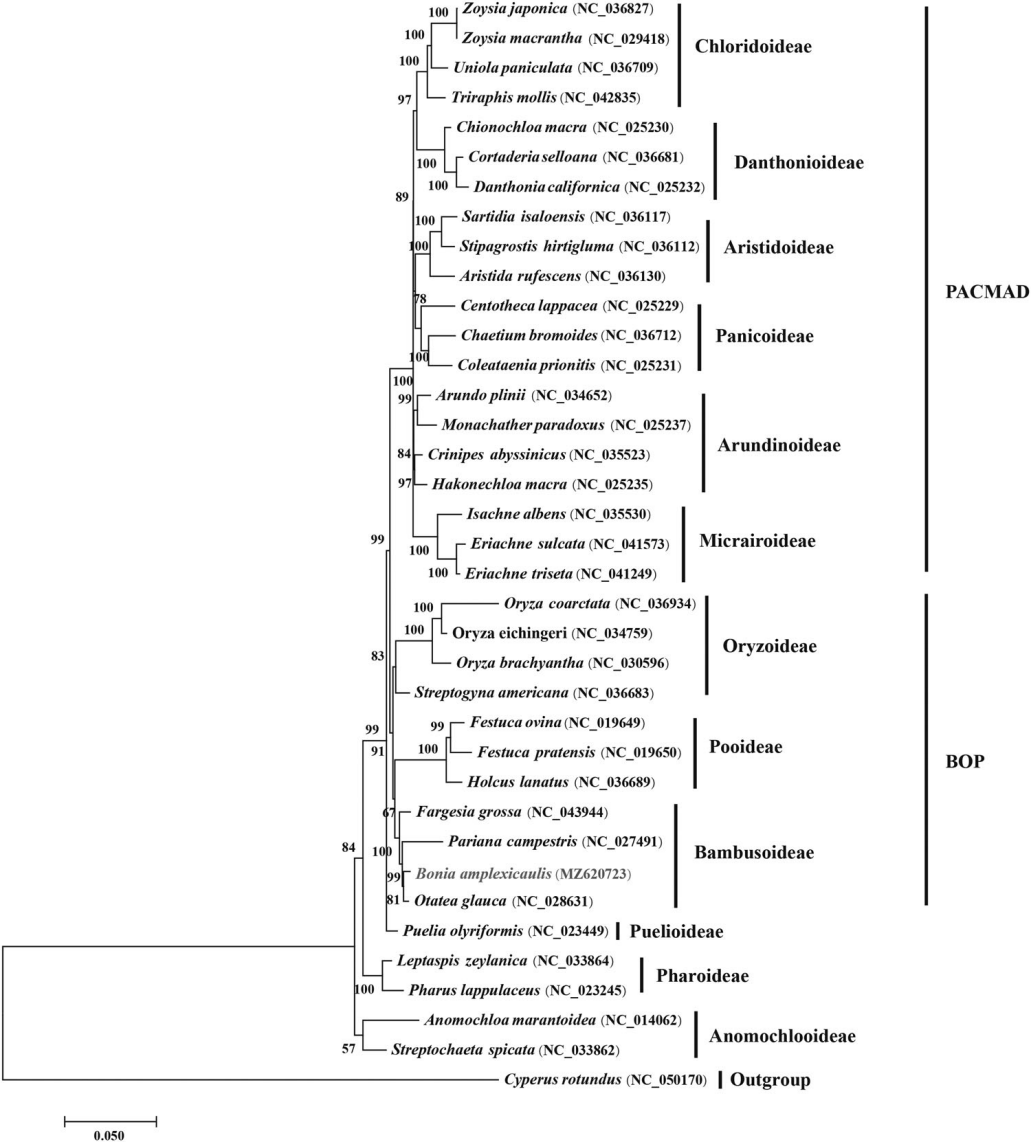
Maximum likelihood phylogenetic tree based on all protein-coding genes of the 36 grass complete chloroplast genomes using *Cyperus rotundus* as outgroup. Bootstraps values (1000 replicates) are shown at the nodes.

## Data Availability

The genome sequence data that support the findings of this study are openly available in GenBank of NCBI at [https://www.ncbi.nlm.nih.gov] (https://www.ncbi.nlm.nih.gov/) under the accession no. MZ620723. The associated BioProject, SRA, and Bio-Sample numbers are PRJNA744348, SRR15096927, and SAMN20130507 respectively. The data that newly obtained at this study are also publicly available in the National Genomics Data Center at https://ngdc.cncb.ac.cn under the accession number of GWHBCKJ00000000.
